# The ArcB Leucine Zipper Domain Is Required for Proper ArcB Signaling

**DOI:** 10.1371/journal.pone.0038187

**Published:** 2012-05-30

**Authors:** Luis Alberto Nuñez Oreza, Adrián F. Alvarez, Imilla I. Arias-Olguín, Alfredo Torres Larios, Dimitris Georgellis

**Affiliations:** 1 Departamento de Genética Molecular, Instituto de Fisiología Celular, Universidad Nacional Autónoma de México, México, D.F., México; 2 Departamento de Fisiología, Facultad de Medicina, Universidad Nacional Autónoma de México, México, D.F., México; 3 Departamento de Bioquímica y Biología Estructural, Instituto de Fisiología Celular, Universidad Nacional Autónoma de México, México, D.F., México; Universidad Nacional Autonoma de Mexico, Instituto de Biotecnologia, Mexico

## Abstract

The Arc two-component system modulates the expression of numerous genes in response to respiratory growth conditions. This system comprises ArcA as the response regulator and ArcB as the sensor kinase. ArcB is a tripartite histidine kinase whose activity is regulated by the oxidation of two cytosol-located redox-active cysteine residues that participate in intermolecular disulfide bond formation. Here, we report that the ArcB protein segment covering residues 70–121, fulfills the molecular characteristics of a leucine zipper containing coiled coil structure. Also, mutational analyses of this segment reveal three different phenotypical effects to be distributed along the coiled coil structure of ArcB, demonstrating that this motif is essential for proper ArcB signaling.

## Introduction

**Figure 1:**
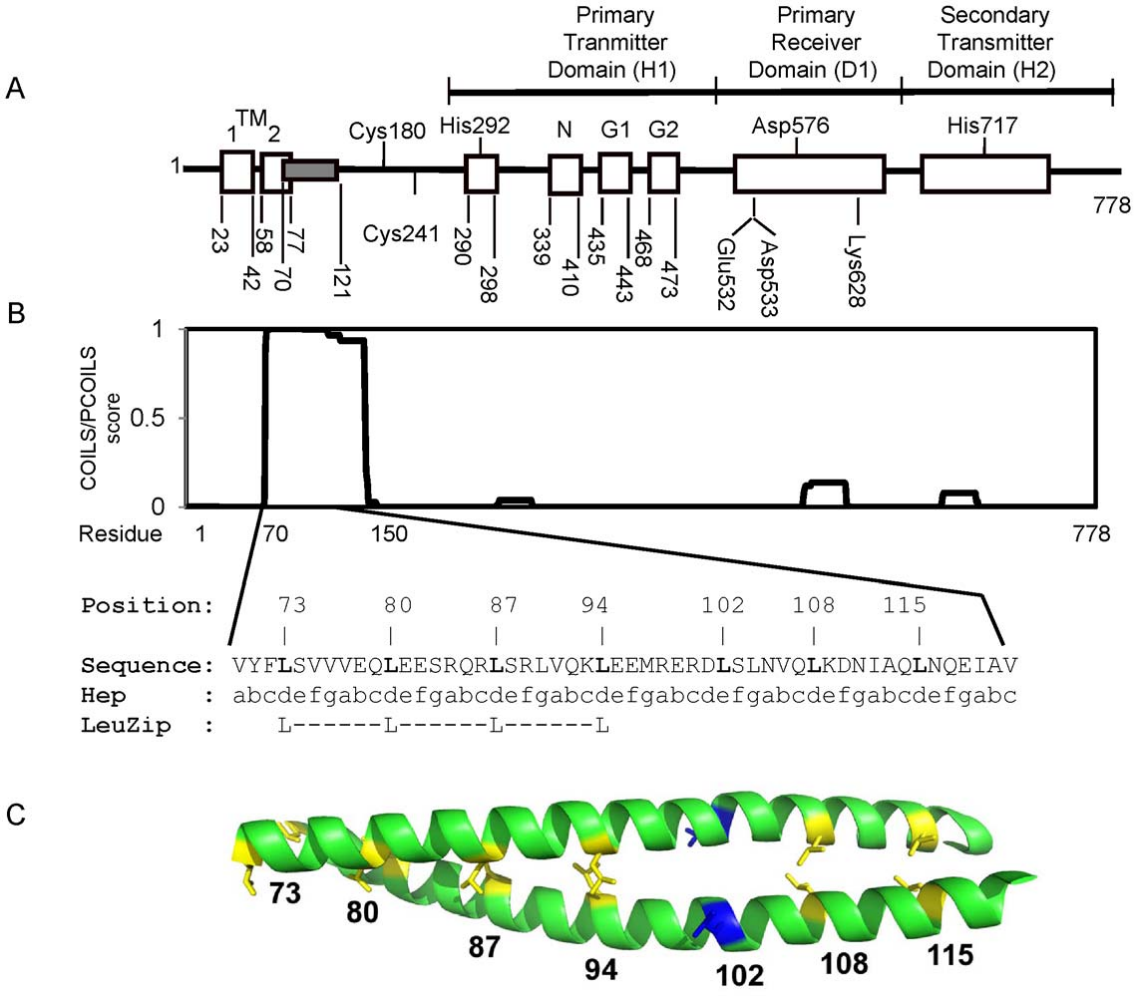
Schematic representation of domain composition in ArcB and coiled coil **prediction.** **A**) The ArcB sensor kinase is attached to the plasma membrane by TM1 corresponding to residues 23 to 41 and TM2 corresponding to residues 58 to 77. The linker region contains a coiled coil motif with a putative leucine-zipper (gray filled block) and two redox-active cysteine residues 180 and 241. The primary transmitter domain (H1) contains the conserved His292 and the catalytic determinants N, G1, and G2. The G1 and G2 sequences typify nucleotide-binding motifs. The receiver domain (D1) contains the conserved Asp576, and the histidine phosphotransfer domain (Hpt/H2) contains the conserved His717. Adapted from [7] but with some modifications. **B**) Prediction of coiled coil motifs along the ArcB amino acid sequence. Above is plotted the probability of coiled coil structure occurrence along the ArcB sequence, as predicted by the COILS/PCOILS tool. Below is presented the sequence of the ArcB coiled-coil motif with its heptad repeats, and the leucine zipper as predicted by the program 2ZIP. **C**) Model of the ArcB coiled coil section covering residues 73–121. The structure prediction was generated based on the crystal structure of a model dimeric coiled coil (PDB code 3he5). The positions of leucine residues 73, 80, 87, 94, 108 and 115 are indicated in yellow and the position of Leu 102, which was used as an experimental control, is shown in blue.

The Arc (anoxic redox control) two-component system plays an important role in the complex transcriptional regulatory network that allows facultative anaerobic bacteria, such as *Escherichia coli*, to sense changes in respiratory growth conditions and adapt their gene expression accordingly [1]–[3]. This system consists of ArcB as the sensor kinase and ArcA as the response regulator [4], [5]. The ArcB protein belongs to a subfamily of tripartite hybrid kinases; in addition to the canonical pair of transmembrane segments and the orthodox transmitter domain (H1), it possesses a central receiver domain (D1) and a secondary C-terminal transmitter domain (H2 or HPt) [5], [6] (Fig. 1A). Moreover, in the linker region, that is the region connecting the catalytic domains with the transmembrane domain, there is a putative leucine zipper [7] and a PAS domain [8].

Under reducing conditions, ArcB autophosphorylates through an intramolecular reaction [9], a process that is enhanced by anaerobic metabolites such as D-lactate and acetate [10], [11], and transphorylates ArcA via a His292 → Asp576 → His717 → Asp54 phosphorelay [12], [13]. Phosphorylated ArcA represses the expression of many operons involved in respiratory metabolism, and activates a few operons encoding proteins involved in fermentative metabolism and micro-aerobic respiration [14]–[17]. Under non-stimulating conditions ArcB acts as a phosphatase that catalyzes the dephosphorylation of ArcA-P by a reverse Asp54 → His717 → Asp576 → Pi phosphorelay [7], [18].

Previously, it was reported that regulation of the catalytic activity of ArcB is set by rotational movements that alter the orientation of the cytosolic portion of ArcB [19], and that the molecular event for ArcB regulation involves the oxidation of two cytosol-located redox-active cysteine residues that participate in intermolecular disulfide bond formation, a reaction in which quinones act as direct oxidants [20], [21]. The evolution of such a mechanism, involving intermolecular disulfide bond formation, would require that the ArcB protein evolved to operate as a homo-dimer, in order to promote the proximity of the two cysteine-residues. Although, it is generally accepted that the conserved transmitter domain of sensor kinases may promote dimerization of these proteins [22], the existence of a putative leucine zipper-like motif in the linker region of ArcB [7], and the fact that leucine zipper motifs have been implicated in homo- and hetero- dimer formation of various proteins, prompted us to hypothesize that this motif may also act as a dimerization domain for the ArcB protein. It has to be noted that in a previous study addressing this question, it was concluded that this putative leucine zipper is not a functional motif [23]. However, the results presented here indicate that the putative leucine zipper motif in the linker region of ArcB [7] fulfills the molecular characteristics of a *bona fide* leucine zipper, and that it’s structural integrity is required for proper regulation of ArcB activity.

## Results

### Sequence Analysis of the ArcB Linker Region Suggests a Coiled Coil Fold that Fulfills the Theoretical Characteristics of a Leucine Zipper

The transmembrane domain of ArcB is immediately followed by a stretch of amino acids, which appears to have a feature characteristic of the well-documented leucine zipper motif [24]. The diagnostic feature of this motif has been defined as an amphipathic helix with hydrophobic residues clustered on one face and hydrophilic residues on the opposite face, and a leucine residue at the first position in each of four contiguous heptad-repeats (LX_6_LX_6_LX_6_LX_6_). In each of these coiled coil heptad-repeats that are denoted “abcdefg”, positions “a” and “d” are occupied by non-polar residues, having the leucine in position “d”, whereas positions “e” and “g” are occupied by polar residues that are solvent-exposed [25]. It is clear from previous studies that such a motif is involved in homo- or hetero-dimer formation through interaction of the helices from two monomers, via their parallel hydrophobic faces, to give a coiled coil dimeric structure [26]. Computer-aided analysis of the secondary structure of ArcB, using the program 2zip [27], revealed that the proposed protein section fulfills the characteristics of this well-documented motif, having the conserved leucine residues at position 73, 80, 87, and 94 (Fig. 1B). Moreover, the COILS/PCOILS prediction method [28] suggested that the coiled coil structure continues to amino acid 150 with a significant score (Fig. 1B), having the leucines 108 and 115 in the same face of the helix as the ones of the upstream leucine zipper. In Fig. 1C it is presented a structural model of the dimeric coiled coil motif of ArcB, where the proximity of the hydrophobic chains of leucine 73, 80, 87, 94, 108 and 115, but not of leucine 102 is indicated. In general, leucine zippers are found in DNA-binding regulatory proteins [29], but are also present in membrane proteins that do not bind to DNA [22], [30]–[33].

### The Integrity of the Leucine-zipper is Required for Proper *in vivo* ArcB Signaling

The functionality of the predicted leucine zipper motif in ArcB was explored by constructing a series of low copy number plasmids (pMX 734–748) expressing ArcB proteins in which each of the four leucine residues, or combinations of them, were substituted to valine. Valine was chosen because it provides the most moderate amino acid substitution, producing a weakness of the coiled coil bundle without disrupting the helicity of this region of the protein.

**Figure 2:**
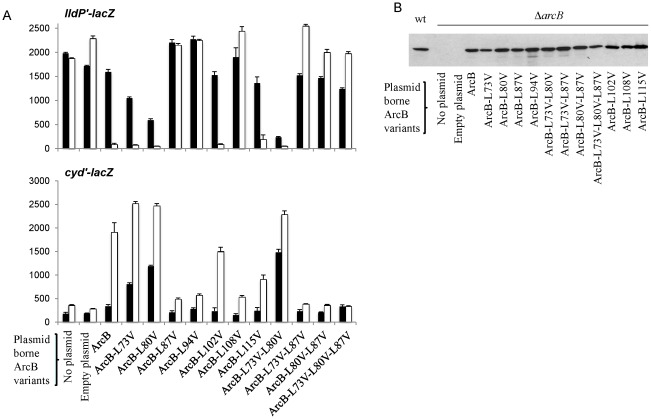
Effect of mutations in the leucine zipper of ArcB on the expressions of λΦ(*cydA’-lacZ*) and λΦ(*lldP’- lacZ*) operon fusions. **A**) Strains ECL5012 [λΦ(*lldP’- lacZ*)] and ECL5004 [λΦ(*cydA’-lacZ*)] carrying low copy plasmids that harbor the *arcB* mutant variants were grown aerobically (solid bars) or anaerobically (empty bars) in Luria-Bertani broth containing 0.1 M MOPS (morpholinepropanesulfonic acid; pH 7.4) and 20 mM D-xylose. In the case of the λΦ(*lldP’- lacZ*)-bearing strains the growth medium was supplemented with 20 mM L-lactate as an inducer. At mid-exponential growth phase (OD_600_ ∼ 0.5) the cells were harvested and the ß-Galactosidase activity was assayed and expressed in Miller units. The data are averages from three independent experiments and the standard deviations are indicated. (**B**) Equal number of bacteria of the above aerobic cultures were analyzed by Western blot analysis, using ArcB polyclonal antibodies as previously described [34].

The consequences of ArcB modifications were then analyzed by monitoring the *in vivo* levels of phosphorylated ArcA, as indicated by the expression of the positively controlled *cydA’-lacZ* and negatively controlled *lldP’- lacZ* target operons. To this end, the generated plasmids were transformed into the Δ*arcB* strains ECL5004 and ECL5012, carrying a λΦ(*cydA’-lacZ*) operon fusion and a λΦ(*lldP’- lacZ*) operon fusion, respectively [34]. All plasmid born *arcB* alleles produced wild-type levels of ArcB (Fig. 2B), as judged by Western blot analysis of the cell extracts with polyclonal antiserum raised against purified His_6_-ArcB^78–520^ (Fig. 2B), indicating that the L to V substitutions do not affect the stability of the mutant proteins.

The transformants were grown aerobically or anaerobically in buffered Luria-Bertani broth (LB) to an OD600 of ∼ 0.5, and their ß-galactosidase activity was determined (Fig. 2A). It was found that substitution of L73V resulted in a 2-fold lower *lldP-lacZ* expression and a 2.8-fold higher *cyd-lacZ* expression under aerobic growth, indicating a partially constitutive active ArcB. Likewise, substitution L80V, having a more drastic effect, showed an almost 3-fold lower *lldP-lacZ* expression and 3.5-fold higher *cyd-lacZ* expression. Moreover, this aerobic activity was more profound in the double L73V-L80V substitution, showing a 7-fold lower *lldP-lacZ* expression and a 4.3-fold higher *cyd-lacZ* expression. However, no significant difference was observed on the anaerobic ß-galactosidase activities, indicating that these ArcB mutants are still subjected to redox regulation. On the other hand, substituting L87 or L94 to V, and all combination mutants involving either of L87V or L94V resulted in an *arcB* null phenotype (Fig. 2, and data not shown). It has to be mentioned that in a previous study it was reported that substitution of L94 to A, in contrast to the L94 to V mutant, resulted in a wild type ArcB activity [23]. In an attempt to confirm this result, the effect of substituting either of L73, L80, L87 and L94 to A on the activity of ArcB was tested. All L to A mutants had the same effect as the L to V mutants on the activity of ArcB, except the L94 to A, which in accordance to the previous report, resulted in a wild type ArcB activity (data not shown).

As mentioned earlier, L108 and L115 are predicted to be in the same face of the helix as the ones of the upstream leucine zipper. To test whether these leucine residues are of importance for the activity/regulation of ArcB, they were substituted to valine and their aerobic and anaerobic reporter expression was monitored (Fig. 2). It was found that the L108V mutation rendered ArcB inactive, whereas the L115V mutation exhibited lower anaerobic ArcB activity. Finally, substitution of L102 to V was used as a control, because it was predicted to not be in the same face of the helix as the ones of the upstream leucine zipper. As expected this mutant exhibited almost wild type ArcB activity.

### The Integrity of the Leucine-zipper is Required for Proper *in vitro* ArcB Activity

It has been previously shown that the kinase activity of ArcB is inhibited by ubiquinone-0 (Q0, a soluble analog of ubiquinone-8) [20]. We, therefore, tested the effect of Q0 on the activity of the wild type ArcB, and the mutant ArcB^L80V^ and ArcB^L87V^ proteins in isolated membrane vesicles containing high amounts of either the three ArcB variant proteins. ArcB^L80V^ and ArcB^L87V^ were selected as representatives for mutants with increased or no ArcB activity, respectively.

**Figure 3:**
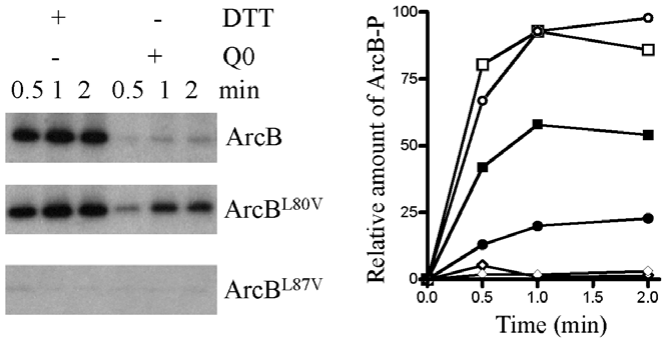
Effect of DTT and ubiquinone-0 on the rate of ArcB net-phosphorylation. Membrane vesicles (1 µg) containing high amounts of wild type ArcB^1–778^ (circles) or the mutant ArcB variants (ArcB^L80V^ (squares) and ArcB^L87V^ (diamonds)) were incubated at room temperature with 40 µM [γ-^32^P]ATP in the presence of 5 mM DTT (open symbols) or 250 µM Q0 (closed symbols) in a 20 µl reaction mixture. At the indicated time intervals a 5 µl sample was withdrawn for SDS-PAGE analysis. Left panel: autoradiograms of the gels. Right panel: net increase of ArcB-P with time, as quantitated with a PhosphorImager.

SDS-PAGE analysis of the membrane fractions followed by Coomassie Blue staining revealed that ArcB was the major component in the membrane preparations (data not shown). Subsequently, 1 µg of total protein (membrane vesicles), carrying either the wild type or the mutant ArcB proteins, were incubated with [γ-^32^P]ATP in the presence of dithiothreitol (DTT) or Q0, as described previously [20]. In agreement with the previous report using a truncated form of ArcB (ArcB^78–778^), the full length ArcB protein was rapidly phosphorylated in the presence of DTT, and its phosphorylation was inhibited in the presence of Q0 (Fig. 3), indicating that the over-expressed protein in the membrane fractions preserves its activity and regulation. However, although the net-phosphorylation kinetics of the ArcB^L80V^ mutant were similar to the one of the wild type ArcB in the presence of DTT, the ArcB^L80V^ mutant was significantly more resistant to Q0-dependent inhibition than the wild type ArcB protein (Fig. 3). Thus, in agreement with the *in vivo* results, the L80V substitution renders the protein partially insensitive to Q0. On the other hand, no phosphorylation was observed for the ArcB^L87V^ mutant (Fig. 3), indicating that the L87V mutation renders the protein inactive.

### The ArcB Mutants Exhibit a Dominant Negative Phenotype

**Figure 4:**
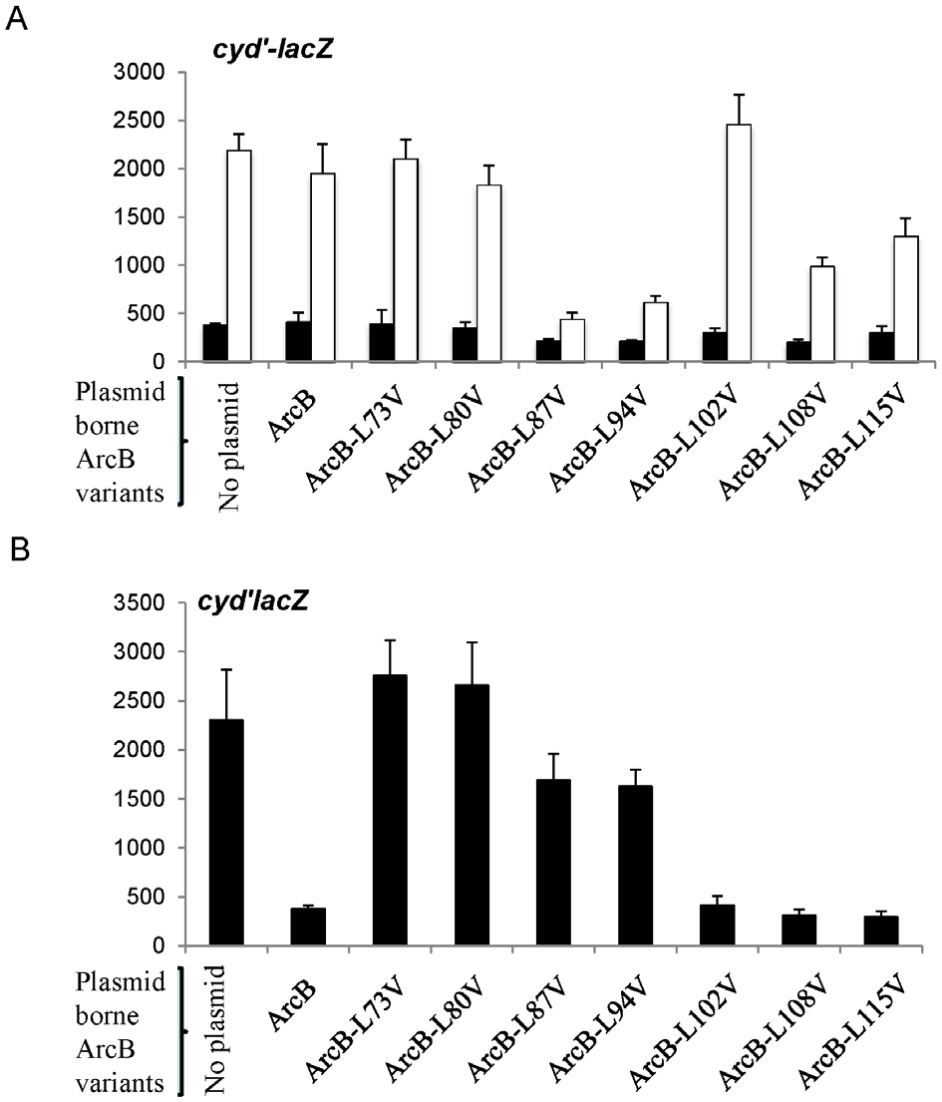
Effect of mutations in the leucine zipper of ArcB on its phosphatase activity and their dominant phenotypes. **A**) Strain ECL5003 [*arcB* ^wt^, λΦ(*cydA’-lacZ*)] carrying low copy plasmids with the *arcB* mutant variants was grown aerobically (solid bars) or anaerobically (empty bars) to mid-exponential growth phase (OD_600_ ∼ 0.5), and the ß-Galactosidase activity was assayed and expressed in Miller units. The data are averages from three independent experiments and the standard deviations are indicated. **B**) Effect of Leu replacements on the phosphatase activity of ArcB. Strain ECL5004, transformed with low-copy plasmids carrying different *arcB* forms, was grown aerobically to mid-exponential growth phase (OD_600_ ∼ 0.5), on minimal medium with pyruvate as sole carbon source, and ß-Galactosidase activity was assayed and expressed in Miller units. The data are averages from three independent experiments and the standard deviations are indicated.

Because the above described *in vivo* and *in vitro* analyses of the leucine-zipper ArcB mutants revealed either a semi-constitutive ArcB kinase (L73V and L80V) or an inactive ArcB kinase (L87V and L94V), we asked whether these phenotypes are dominant in a wild-type strain. To this end, the *arcB^+^* strain ECL5003 was transformed with the mutant ArcB expressing plasmids, and the expression of the λΦ(*cydA’-lacZ*) operon fusion was monitored. The transformants were grown aerobically or anaerobically in buffered Luria-Bertani broth (LB) to an OD600 of ∼ 0.5, and their ß-galactosidase activities were determined (Fig. 4A). It was found that the *arcB* ^+^ strain carrying either the L87V or the L94V ArcB mutant expressing plasmids failed to activate *cydA’-lacZ* expression under anaerobic conditions (Fig. 4A). Also, expression of the plasmid borne L108V and the L115V ArcB mutants in an *arcB* ^+^ strain resulted, respectively, in a 2.2-fold and a 1.5-fold reduction of *cydA’-lacZ* expression under anaerobic conditions (Fig. 4A).

On the other hand, the substitution L73V and L80V that exhibit a semi-constitutive ArcB activity, and also the substitution L102V, which was used as a control, had no effect on the *cydA’-lacZ* expression under the above mentioned conditions (Fig. 4A). It thus appears that the L to V substitutions that result to either lower or no ArcB kinase activity, cause a dominant negative phenotype.

### The Dominant Negative Phenotype of the Leucine Zipper Mutants is Not Due to the Phosphatase Activity of ArcB

The above results led us to consider the possibility that the inactive ArcB variants could be trapped in a kinase^−^/phosphatase^+^ state, counteracting the kinase activity of the chromosomally expressed wild type ArcB. To test this possibility, we generated ArcB-independent ArcA-P *in vivo*, and examined whether the ArcB mutant variants were able to dephosphorylate ArcA-P [18]. To this end, the ECL5004 strain (Δ*arcB*, *cydA’-lacZ*) harboring the low copy number plasmids expressing either the wild type ArcB or the various ArcB mutants, was grown aerobically in defined minimal medium supplemented with 20 mM pyruvate as the sole carbon and energy source, to an OD600 of ∼ 0.5, and the ß-galactosidase activity was determined. Growth on pyruvate was chosen because it has been shown that under this condition the intracellular concentration of acetyl-phosphate is an order of magnitude higher than in cells grown aerobically on glycerol as the sole carbon and energy source [35], and because in the absence of their cognate sensor kinase, many response regulators undergo *in vivo* autophosphorylation at the expense of acetyl-phosphate [36]–[39]. In agreement with a previous report [18], it was found that the *cydA’-lacZ* reporter was suitably expressed in the Δ*arcB* strain, indicating a deficiency in ArcA-P dephosphorylating activity, whereas reporter expression was 6-fold lower in the strain carrying the wild type ArcB expressing plasmid (pMX732), indicating specific ArcA-P dephosphorylation by ArcB under aerobic growth conditions (Fig. 4B). Moreover, it was found that the L73V and L80V semi-constitutive ArcB mutants exhibited almost the same level of reporter expression as the Δ*arcB* strain, indicating a complete loss of phosphatase activity. On the other hand, the L87V and L94V ArcB mutants resulted in approximately 1,4-fold lower reporter expression than the one observed in the Δ*arcB* mutant strain, and ∼4.5-fold higher reporter expression than the wild type ArcB, indicating not only loss of kinase activity (Fig. 2) but also a severe deficiency in their phosphatase activity under aerobic growth. Thus, the possibility of an ArcB conformation trapped in a kinase^−^/phosphatase^+^ state can be discarded. Instead, formation of heterodimers between chromosomally expressed wild type ArcB and plasmid borne inactive ArcB proteins, could provide a possible explanation for the above result. Finally, the L102V substitution resulted in reporter expression levels similar to those of the wild type ArcB, whereas L108V and L115V mutations resulted to a slight lower reporter expression than the wild type ArcB, indicating that these two latter mutants might have higher phosphatase activity than the wild type ArcB.

## Discussion

Coiled coils are common structural motifs, resulting from the packing of two to five α-helices, one wrapped around the other into a left-handed helix, to form a supercoil [40]. Each helix consists of multiple copies of a heptad-repeating unit (denoted abcdefg), containing a similar configuration of residues [41]. Coiled coil motifs are often found in sensor kinases and in many cases they have been shown to play a significant role in the signaling mechanisms. For example, the HAMP domain, which is present in ∼31% of the sensor kinases, is usually located immediately after the transmembrane region and is of main importance for signal transmission [42]. Another common functional coiled coil motif in sensor kinases is the signaling helix (S-helix), which was suggested to function as a switch that prevents constitutive activation of downstream signaling domains [43]. Leucine zippers constitute a subtype of coiled coil structures, in which the amino acid leucine is predominant at the “d” position of the heptad repeat [24]. Although leucine zippers have been best characterized in DNA binding proteins, they also exist in many other signaling proteins [30]–[33]. Recently, it was reported that the leucine zipper of the cell-cycle regulated Nek2 kinase is important for its dimerization and activation [44].

The fact that the regulation of ArcB signaling involves intermolecular disulfide-bonds formation between two ArcB proteins, suggests that the evolution of such a mechanism would require adaptation of the protein to function as a dimer to promote the proximity of the two cysteine residues. A suitable candidate to promote such a dimer-formation in the linker region of ArcB appears to be the leucine zipper. Accordingly, substitution to valine of any of the leucine residues in this coiled coil motif produced a mutant phenotype. Interestingly, substitution of the first two leucine residues, L73 and L80, resulted in a semi-constitutive ArcB kinase with concomitant loss of its ArcA-P dephosphorylating activity. Thus, the weakness of the hydrophobic helix-helix interaction by the L73V and L80V substitutions may affect the downstream conformation in such a manner that it partially traps the protein in the kinase conformation, which is reminiscent to the effect of mutating the interaction region of the *E. coli* chemoreceptors Tar and Tsr [45]. On the other hand, alterations of the second half of the leucine zipper, L87 and L94, resulted in an inactive ArcB protein, not only as kinase but also as a phosphatase. Finally, a third phenotypical group is formed by L108 and L115. While the kinase activity of ArcB was totally and partially lost by the L108 and L115 substitutions to valine, the phosphatase activity of these mutants was not affected. Even though it is difficult to directly determine the effects of a given “knob” truncation on helix packing stability, the three different phenotypical effects are distributed along the coiled coil structure of ArcB, from the second TM region to the redox active cysteine residues.

Noteworthy, in a previous study, based on *in vivo* and *in vitro* experiments, it was suggested that the leucine zipper of ArcB is not a functional motif. This conclusion was based on the fact that the L94A substitution was without effect, a result confirmed in our study (data not shown), but overlooking the fact that a L80A substitution showed a semi-constitutive kinase activity and a L87A substitution resulted in an *arcB* null phenotype [23]. Although the reasons of the difference between the L94V and L94A substitution are not clear, the higher capability of alanines in comparison to valines to form an α-helix could provide a suitable explanation [46]. Interestingly, the L to V substitutions, resulting in null or lower ArcB kinase activity, were shown to exhibit a dominant phenotype when expressed in an *arcB* ^+^ strain. Such an effect could occur if the ArcB mutants were trapped in a kinase^−^/phosphatase^+^ conformation, which appears to be the case of the ArcB^L108V^ and ArcB^L115V^ mutants, thereby counteracting the kinase activity of the chromosomal encoded ArcB protein. On the other hand, heterodimer formation between the chromosomal encoded ArcB protein and the inactive ArcB variants, could provide a possible explanation for the dominant negative phenotype of the ArcB^L87V^ and ArcB^L94V^ mutants.

Taken together, our experimental results clearly indicate that the structural integrity of not only the leucine zipper but also the overall coiled coil fold of ArcB is essential for the correct orientation of the monomers within a dimer, and thereby required for proper ArcB signaling.

## Materials and Methods

### Bacterial Strains, Plasmids and Oligonucleotides

*Escherichia coli* strains and plasmids used in this study are listed in Table 1. Plasmid pMX712 was constructed by cloning the *Bam*HI-*Hin*dIII fragment from plasmid pIBW [34], which carries the *arcB* promoter, the *arcB* ribosome binding site, an introduced *Nde*I site that includes the initiation codon of *arcB*, and the *arcB* ORF and stop codon, into pBlueScript II KS^+^. Plasmids pMX734-737 and pMX528-530 were constructed by site-directed mutagenesis of plasmid pMX712, substituting either of leucine 73, 80, 87, 94, 102, 108 or 115 to valine and subsequent cloning of the resulting modified *arcB* gene into plasmid pEXT22. For example, to construct the plasmid expressing ArcB^L73V^, primers 5′-CCCGGATCCCATATGAAGCAAATTCGTCTGCTGGCGC-3′ and 5′-GACGACCACCGACACAAAGTAGACCGC-3′ were used in PCR with plasmid pMX712 as template. The product of this reaction was purified and used as a megaprimer in a PCR together with primer 5′-CTCAGACCGACGATACCGTT-3′ and pMX712 as template. The product of this second PCR was digested with *Nde*I and *Nru*I, and used to substitute the *Nde*I - *Nru*I wild-type fragment of pMX712. Finally, the *Bam*HI - *Hin*dIII fragment of the resultant plasmid was inserted into the *Bam*HI and *Hin*dIII sites of the one-copy number plasmid pEXT22 [47] to generate plasmid pMX734. A similar strategy was employed to generate the other single mutants but with the corresponding mutagenic primer (Leu 80∶5′-CGTGACTCCTCCACTTGCTCGAGC-3′, Leu 87∶5′-CAGCCGTGACACACGTTGTCG-3′, Leu 94∶5′-GCGCATCTCCTCCACTTTTTGCACCAGCCG-3′, Leu 102∶5′-CTGAACGTTGAGGCTCACATCGCGCTCGCGC-3′, Leu 108∶5′-GCTGGGCAATATTATCTTTTACCTGAACGTTGAGGCTC-3′, Leu 115∶5′-CGAACGGCAATTTCCTGATTTACCTGGGCAATATTATC-3′). To construct plasmids with double, triple or quadruple mutations in the leucine zipper we employed the same strategy but with single, double or triple mutant plasmids as the template. All plasmids were verified by DNA sequencing. Plasmids pMX517, pMX520 and pMX521 were constructed by substituting the *Nde*I*-Hin*dIII fragment of pMX020 [18] with the *Nde*I*-Hin*dIII fragment of plasmids pMX712 (wild type ArcB), pMX715 (ArcB^L80V^), and pMX716 (ArcB^L87V^).

**Table 1:** *Escherichia coli* strains and plasmids used in this study are listed.

Strain		Relevant characteristics	Source	
ECL5004		Δ*arcB*::Tet^r^ λФ(*cydA*’*-lacZ*) Δ*fnr*::Tn*9*(Cm^r^)	[34]	
ECL5012		Δ*arcB*::Tet^r^ λФ(*lldP*’*-lacZ*)	[34]	
ECL5003		*arcB* ^+^ λФ(*cydA*’*-lacZ*) Δ*fnr*::Tn*9*(Cm^r^)	[34]	
**Plasmids**				
pBluescript SK II (+)	Cloning vector, Amp^r^			Stratagene
pEXT22	Low copy number vector, Kan^r^			[47]
pMX712	*arcB* ^+^ in pBluescript KS II (+)			This study
pMX715	*arcB* ^L80V^ in pBluescript KS II (+)			This study
pMX716	*arcB* ^L87V^ in pBluescript KS II (+)			This study
pMX732	*arcB* ^+^ in pEXT22			This study
pMX734	*arcB* ^L73V^ in pEXT22			This study
pMX735	*arcB* ^L80V^ in pEXT22			This study
pMX736	*arcB* ^L87V^ in pEXT22			This study
pMX737	*arcB* ^L94V^ *in pEXT22*			This study
pMX528	*arcB* ^L102V^ in pEXT22			This study
pMX529	*arcB* ^L108V^ in pEXT22			This study
pMX530	*arcB* ^L115V^ in pEXT22			This study
pMX738	*arcB* ^L73V-L80V^ in pEXT22			This study
pMX739	*arcB* ^L73V-L87V^ in pEXT22			This study
pMX741	*arcB* ^L80V-L87V^ in pEXT22			This study
pMX744	*arcB* ^L73V-L80V-L87V^ in pEXT22			This study
pMX020	*arcB* ^521–778^ under control of *ara* promoter, Amp^r^			[18]
pMX517	*arcB* ^WT^ under control of *ara* promoter, Amp^r^			This study
pMX520	*arcB* ^L80V^ under control of *ara* promoter, Amp^r^			This study
pMX521	*arcB* ^L87V^ under control of *ara* promoter, Amp^r^			This study

### Growth Conditions

*Escherichia coli* strains were routinely grown in Luria-Bertani (LB) medium at 37°C. When necessary, ampicillin, kanamycin, tetracycline or chloramphenicol was used at final concentrations of 100, 50, 12.5 or 34 µg ml^-1^, respectively. To induce expression of the *ara* promoter controlled genes, arabinose was added to a final concentration of 0.13 mM. To estimate *in vivo* phosphatase activity of the ArcB variants, ArcB-independent ArcA-P was generated by growing cells in a defined minimal medium [1 mM KH_2_PO_4_, 40 mM KCl, 34 mM NaCl, 20 mM (NH_4_)_2_SO_4_, 1 µM FeSO_4_, 0.3 mM MgSO_4_, 1 µM ZnCl_2_, 10 µM CaCl_2_, and 0.1 M MOPS, at a final pH of 7.4] supplemented with 20 mM pyruvate as described previously [9].

### ArcB-enriched Inverted Vesicles Preparation and Phosphorylation Assays

Strain ECL5012 carrying pMX517, pMX520 or pMX521, was grown in 250 ml of ampicillin containing LB medium at 37°C until an OD_600_ of 0.5. Then, expression of ArcB was induced by addition of L-arabinose to a final concentration of 0.13 mM. Cells were harvested 4 hours after induction, resuspended in 6 ml of ice-cold MOPS buffer (50 mM K-MOPS, pH 7.0, 5 mM MgSO_4_, and 100 mM KCl), and broken by passing through a French Press. The cell lysate was cleared by centrifugation at 10,000 g for 15 min, and the ArcB embedded into inner membranes was centrifuged at 32,500 *g* for 40 min at 4°C. Finally, the membranes were solubilized in 500 µl of MOPS buffer containing 30% glycerol, and stored at −20 °C. Phosphorylation assays were carried out at room temperature in the presence of 40 mM [γ-^32^P]ATP (specific activity, 2 Ci mmol^−1^; New England Nuclear), 33 mM HEPES (pH 7.5), 50 mM KCl, 5 mM MgCl_2_, 0.1 mM EDTA, and 10% glycerol. 1 µg of the total protein (membrane vesicles), carrying either the wild type or the mutant ArcB proteins, was used in phosphorylation assays in the presence of 5 mM dithiothreitol (DTT) or 250 mM Q0, as described previously [48].

### β-Galactosidase Activity Assay

The λΦ(*cydA’-lacZ*) bearing strains were grown in Luria-Bertani broth containing 0.1 M MOPS (morpholinepropanesulfonic acid; pH 7.4) and 20 mM D-xylose. The λΦ(*lldP’- lacZ*)-bearing strains were grown in the above medium supplemented with 20 mM L-lactate as an inducer. For aerobic growth, cells were cultured in 10 ml of medium in 250-ml baffled flasks at 37°C with shaking (300 rpm). For anaerobic growth, cells were cultured in a screw-capped tube filled with medium up to the rim at 37°C and stirred by a magnet. β-Galactosidase activity was assayed with exponentially growing cultures and expressed in Miller units as described previously [49].

### Western Blot Analysis

Aerobically grown cultures were harvested by centrifugation during mid-exponential growth. The cell pellet was resuspended in 5X- SDS sample buffer and separated by SDS-PAGE (10% polyacrylamide gel), and the proteins were transferred to a Hybond-ECL filter (Amersham Biosciences). The filter was equilibrated in TTBS buffer (25 mM Tris, 150 mM NaCl, and 0.05% Tween 20) for 10 min and incubated in blocking buffer (1% milk in TTBS) for 1 h at room temperature. ArcB polyclonal antibodies, raised against His_6_-ArcB^78–520^ [12], were added at a dilution of 1∶10,000 to the filter and incubated for 1 h at room temperature. The bound antibody was detected by using anti-rabbit IgG antibody conjugated to horseradish peroxidase and the ECL detection system (Amersham Biosciences).

### Structural Model of the ArcB Coiled Coil Linker Region

The structural model was inferred using the results, with the highest normalized Z-score, provided by the I-TASSER server (http://zhanglab.ccmb.med.umich.edu/I-TASSER/) [50]. The predicted model is based on the monomeric helix (covering amino acid residues 70–89) derived from the solution structure of the ArcB transmembrane domain (PDB code 2 ksd), and using the original coordinates of the dimeric coiled coil motif from PDB code 3he5 as a template.
